# Solute Clearance Evaluation and Filter Clotting Prediction in Continuous Renal Replacement Therapy

**DOI:** 10.3390/jcm12247703

**Published:** 2023-12-15

**Authors:** Kohei Yoshimoto, Ryo Matsuura, Yohei Komaru, Teruhiko Yoshida, Yoshihisa Miyamoto, Yoshifumi Hamasaki, Ryota Inokuchi, Masaomi Nangaku, Kent Doi

**Affiliations:** 1 Department of Emergency and Critical Care Medicine, The University of Tokyo, Tokyo 113-8655, Japan; yoshimotok-icu@h.u-tokyo.ac.jp (K.Y.);; 2 Department of Nephrology and Endocrinology, The University of Tokyo, Tokyo 113-8655, Japanteyoshida-tky@umin.ac.jp (T.Y.);

**Keywords:** clearance, urea, myoglobin, clotting, filter, continuous hemodiafiltration

## Abstract

Unexpected filter clotting is a major problem in continuous renal replacement therapy (CRRT). Reduced solute clearance is observed prior to filter clotting. This single-center, retrospective, observational study aimed to determine whether reduced solute clearance of low- and medium-molecular-weight molecules in CRRT can predict filter clotting. Solute clearances of urea and myoglobin (Mb) were measured at 24 h after initiation of continuous hemodiafiltration (CHDF). Clearance per flow (CL/F) was calculated. The primary outcome was clotting of the filter in the subsequent 24 h, and 775 CHDF treatments conducted on 230 patients for at least 24 consecutive hours in our ICU were analyzed. Filter clotting was observed in 127 treatments involving 39 patients. Urea and Mb CL/F at 24 h were significantly lower in the patients who experienced clotting. Further analysis was limited to the first CHDF treatment of each patient to adjust for confounding factors. Multivariate logistic regression analysis revealed that both urea CL/F < 94% and Mb CL/F < 64% were significant predictors of clotting within the next 24 h. Lower urea and Mb CL/F measured at 24 h after CRRT initiation were associated with filter clotting in the next 24 h. Further study is necessary to ascertain whether measurement of urea and MB CL/F will help with avoiding unexpected filter clotting.

## 1. Introduction

Acute kidney injury (AKI) is one of the most frequently encountered organ injuries that develop in critical care settings. Several clinical studies have previously reported that AKI is a significant and independent predictor of mortality among ICU patients [1]. If renal replacement therapy (RRT) is required for patients suffering from AKI, their mortality rate is unacceptably high [2,3]. Although guidelines do not recommend continuous renal replacement therapy (CRRT) as the first choice of RRT for critically ill patients [4,5], CRRT is considered an effective treatment modality for hemodynamically unstable patients with severe AKI because it enables gentle fluid overload correction and removal of excess uremic toxins [6]. Several studies have reported that CRRT is selected more frequently for dialysis-requiring AKI patients treated in ICUs than IRRT [7].

Solute clearance is known to decrease gradually in CRRT because of reduced membrane permeability resulting from fouling. In addition, solute clearance may be compromised in delivering the prescribed dose due to filter clotting, vascular-access-related problems, and downtime caused by external ICU procedures such as CT scanning and surgery. Unexpected clotting can result in blood loss, increased expenses, and a higher requirement of human resources [8,9]. Appropriate anticoagulation, vascular access, and optimization of CRRT settings are crucial to maintain the patency of an extracorporeal circuit [8]. Thus, monitoring solute clearance and predicting filter clotting are crucial for delivering the exact treatment dose of medication and reducing adverse events associated with CRRT.

The measurement of the solute concentration in the effluent is reportedly effective in assessing filter function [10]. This study found that the fluid and blood urea nitrogen ratio could discriminate compromised filter function with a shorter filter life. Actual clearance of urea during continuous hemodiafiltration (CHDF) was observed to be significantly lower than the estimated clearance based on the prescribed dose, and reduction in filter function with respect to small solute clearance was observed in all the filters over time, even in the absence of any clotting. Among several modalities of CRRT, CHDF is widely used because it offers the combined benefits of diffusion and convection, which allows clearance of low-molecular-weight solutes (<500–1500 Daltons) and medium-molecular-weight solutes (<60,000 Daltons) [11,12]. Evaluation of the clearance of small- and medium-molecular-weight solutes by measuring the urea (60 Daltons) and myoglobin (Mb) (17,200 Daltons) in the effluent is necessary for evaluating filter function, both for dialysis and for filtration. This study aimed to determine whether reduced solute clearance of low- and medium-molecular-weight molecules in CHDF could predict filter clotting in advance.

## 2. Materials and Methods

### 2.1. Design and Study Population

In this retrospective observational study, we routinely measured the effluent urea and myoglobin in the patients treated with CRRT, since 2012, at 24 h after initiation of the process. When the urea clearance and myoglobin clearance per flow rate (described below) were below 50% and 25% of the prescribed dose, respectively, the filters were exchanged. These cutoff values were determined empirically because no data were available when we started to measure these clearances as a clinical routine. Otherwise, filter exchange was considered in the event of increased inlet filter pressure and transmembrane pressure (TMP), together with visual inspection of clotting in the filter and extracorporeal circuit.

All the patients undergoing CHDF in the ICU of the University of Tokyo Hospital since 2012 were screened in this study. Among all these patients, those aged 18 years or older who underwent CHDF in the ICU for at least 24 consecutive hours between 2012 and 2019 were deemed eligible for this study. Patients who had discontinued CHDF within 24–48 h for reasons other than filter clotting, those with missing data, and those with ultrafiltration volumes less than 300 mL/h were excluded from the study. Since several CHDF treatments were provided to each patient, sub-analysis limited to the first treatment in each patient was also conducted.

### 2.2. Data Collection and Measurement

Clinical data were extracted from the medical records. The endpoint of this study was filter clotting, occurring from 24 to 48 h after CHDF initiation that required filter exchange. Necessity of filter exchange due to filter clotting was determined based on a sudden increase in the inlet pressure and TMP, in addition to visual inspection of clotting by the physicians and clinical engineers certified for mechanical device management in Japan.

Solute clearances of urea and Mb were calculated based on their concentrations in the blood and effluent fluid 24 h after CHDF initiation. Blood samples were collected before the filter in the extracorporeal circuit. Urea and Mb were measured at the central laboratory of our hospital via the enzymatic UV-kinetic initial rate method with urease and glutamate dehydrogenase and the latex agglutination method with rabbit anti-human myoglobin antibody, respectively. Since solute clearance is affected by the parameters prescribed for CHDF (dialysis flow rate, replacement flow rate, and ultrafiltration flow rate), we defined clearance per flow (CL/F) as solute clearance divided by flow rate prescription volume × 100. Since urea is excreted both by dialysis and ultrafiltration, CL/F for urea (urea CL/F) was calculated from the sum of the dialysis flow rate and the ultrafiltration flow rate. CL/F for Mb (Mb CL/F) was calculated from the ultrafiltration flow rate only.

### 2.3. CHDF Procedure

CHDF was initiated by the physicians based on the current clinical guidelines [4,5,6]. Vascular catheters were used for all the patients with the right internal jugular vein as the first choice of access vessel and either side of the femoral vein or left internal jugular vein as the second choice. CHDF was performed on all the patients using a ACH-Σ^®^ device (Asahi Kasei Medical Corporation, Tokyo, Japan), with a post-dilution method. Filters were chosen by each physician. As anticoagulant therapy, nafamostat mesylate and unfractionated heparin were used for anticoagulation, and the doses of anticoagulant were adjusted based on the values of activated clotting time (ACT), measured every six hours. The target range of ACT was 160 to 200 s. Adjustment of drug doses was determined by each physician. The flow rates of dialysis, replacement, and ultrafiltration were adjusted in accordance with the physician’s recommendations.

### 2.4. Statistical Analysis

Continuous variables are presented as means ± standard deviation or median (interquartile range), and categorical variables are presented as percentages. Student’s *t*-test or the Mann–Whitney U test was used to compare continuous data. The chi-square test or Fisher’s exact test was used to compare categorical data. A 2-sided value of *p*  <  0.05 was considered statistically significant. The cutoff points for discriminating future clotting at 24–48 h were assessed based on receiver operating characteristic (ROC) curve analysis. The cutoff points at which the Youden index (sensitivity + specificity − 1) [13] was maximized were determined. The association of filter clotting with urea and MB CL/F was investigated by performing multivariate logistic regression analyses, adjusting confounding factors. Only parameters that were significantly associated with filter clotting in univariate linear regression analyses were included in multivariate linear regression models. Statistical analyses were performed using BellCurve for Excel (Social Survey Research Information Co., Ltd., Tokyo, Japan) and JMP Pro software (version 15.0.0; SAS Institute, Cary, NC, USA).

## 3. Results

### 3.1. Filter Clotting from 24 to 48 h after CHDF Initiation

**Figure 1:**
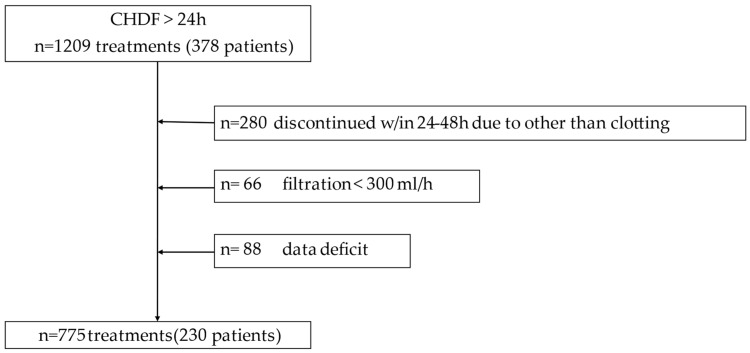
Flow diagram.

During the observation period, we identified 1209 CHDF treatments conducted for more than 24 h on 378 patients, and 775 CHDF treatments conducted on 230 patients were finally included in this study (Figure 1). Filter clotting necessitating filter exchange, occurring from 24 to 48 h after CHDF initiation, was observed in 127 treatments on 39 patients (clotting group), while no filter clotting was observed in 648 treatments on 191 patients (non-clotting group). CHDF prescriptions including types of hemofilter and ACT at 24 h after CHDF initiation are depicted in Table 1. There were no significant differences in the treatment characteristics of CHDF. The ACT was significantly shorter in the clotting group, whereas the dose of nafamostat mesylate was higher in this group. The CL/F of urea and Mb at 24 h was significantly lower in the clotting group. After conducting ROC curve analysis, the area under the curve (AUROC and cutoff points of urea CL/F and Mb CL/F for detecting clotting after 24 h were calculated (Table 2).

### 3.2. Sub-Analysis for the First CHDF Treatment in Each Patient

When all CHDF treatments were included in the analyses, adjustments for individual patient characteristics were difficult, since the number of filters used in each patient was different. Therefore, we limited the analysis to each patient’s first CHDF treatment. The characteristics of the 230 patients and their first CHDF treatments are given in Table 3. The CL/F of urea and Mb at 24 h were significantly lower in the clotting group when limited to the first CHDF treatment in each patient. The AUROC and cutoff points of urea CL/F and Mb CL/F for detecting clotting after 24 h were also calculated in this sub-analysis (Table 4). Multivariable logistic regression analysis revealed that both urea CL/F < 94% and Mb CL/F < 64% were significant predictors of clotting within 24 h after adjusting for possible confounding factors (Table 5). The odds ratio of this combination of CL/F cutoff values was determined as 8.05 [95% confidence interval 2.38–27.3].

## 4. Discussion

Reduced solute clearance during CRRT has been reported previously [10] and is expected to be associated with filter clotting. This study retrospectively evaluated possible associations of urea and MB clearance, measured 24 h after CRRT initiation with subsequent filter clotting within the next 24 h. Significant differences were observed in urea and Mb CL/F measured 24 h after CRRT initiation between the groups that experienced clotting and non-clotting. Further analysis that was limited to the first CRRT in each patient determined the cutoff values of urea and Mb CL/F as 94% and 64%, and the combination of these cutoff values demonstrated a significant odds ratio of 8.0 after adjusting for confounding factors.

Predicting filter clotting in advance is crucial, as filter clotting reduces performance efficiency in CRRT and requires large amounts of medical resources. Several studies have previously examined possible factors predicting filter clotting such as catheter sizes, CRRT modalities, and blood flow rates [14,15]. Parameters of circuit pressure, such as transmembrane pressure (TMP) and inlet or outlet filter pressures, which were obtained hourly have been reported as predictors for circuit clotting [16,17]. Recent technological advances have enabled the continuous monitoring of these pressures, and real-time pressure monitoring enables the prediction of filter clotting [18,19]. However, the reduction in solute clearance has reportedly been observed without the elevation of pressures [10], and increased pressure may in fact suggest the possibility of irreversible clotting. Therefore, evaluation of solute clearance is expected to be a better and faster predictor of clotting than pressure changes. Unfortunately, data on pressure changes are not available in this study. Further studies that compare circuit pressures with solute clearance will be necessary. Anticoagulation strategies certainly have a significant impact on optimizing filter life and subsequently, the performance efficiency of CRRT. Many clinical studies have evaluated optimal anticoagulation strategies, and several meta-analyses have reported favorable effects of citrate over regional heparin in extending filter life and other related outcomes [20,21,22]. The use of citrate as an anticoagulant in RRT is infrequent, since it is considered off-label. Nafamostat mesylate is widely used as an anticoagulant in CRRT for AKI in Japan [12]. Nafamostat mesylate was the commonly used anticoagulant in this study, and the dose was adjusted based on ACT, with a target range of 160–200 s. The possible confounding effect of anticoagulation was adjusted via multiple logistic analysis that incorporated ACT. It is a known fact that in CRRT, nafamostat mesylate increases activated partial thromboplastin time (aPTT). Taken together, the results obtained on clotting prediction using urea and Mb CL/F with cutoff values of 94% and 64% might differ in other CRRT conditions such as citrate use and monitoring of anticoagulant efficacy with aPTT.

In addition, the type of filter membrane might be associated with filter clotting. In this study, two different types of filters (AN69ST membrane and polysurfone) were used based on the physicians’ decisions, and there was a significant difference between the clotting and non-clotting groups in univariate analysis. Two clinical studies have reported that the AN69ST membrane has a negligible effect on the circuit lifespan compared to other membranes [23,24], and multivariable analysis in this study did not indicate any significant impact of membrane type on filter clotting.

Disease conditions are expected to affect the coagulation system and platelet function. Sepsis is known to cause coagulation disorders [25], and many studies have reported a significant association of sepsis with frequent filter clotting in CRRT [26]. A certain proportion of heart failure patients treated using CRRT in ICUs are also treated with antiplatelets and anticoagulants for complicated chronic cardiovascular diseases. The use of systemic heparin in addition to nafamostat mesylate was also observed in this study. Although detailed information was not available in this study, these medications may have some impact on filter clotting in CRRT. In this study, we also adjusted these disease factors and found a significant association between urea and Mb CL/F.

Our study has several limitations that may impact the obtained results. First, this study was performed with a small sample size at a single center, which could restrict the generalizability of our results. Many confounding factors affecting filter clotting in CRRT might not be sufficiently controlled. Of note, the vascular catheter insertion site is known to have a significant impact on filter life [15]. In this study, information on the catheter insertion site was not available. Future studies must be performed with larger cohorts in multicenter ICUs to verify and expand our findings. Second, Mb CL/F cannot reliably reflect the performance of filtration, specifically because some Mb can be removed via dialysis. Although the dialysate flow rate also needs to be considered for Mb CL/F, only the ultrafiltration flow rate was used for Mb CL/F calculation in this study. Another, larger molecule such as β2 microglobulin, which is expelled to a lesser degree by dialysis than Mb, may better reflect the performance of filtration. Third, although CRRT initiation and clinical management were determined for all patients based on recent clinical guidelines [4,5,6], there were no definitive and standardized indicators for CRRT initiation, choice of filters, and dose of dialysis and filtration. Anticoagulation therapy was adjusted based on the ACT values as described in the methods. Since this study was conducted in a retrospective observational manner, future interventional studies should be conducted with a predefined treatment protocol of CRRT including anticoagulation.

## 5. Conclusions

In conclusion, our study found that lower urea and Mb CL/F measured 24 h after CRRT initiation could be used to predict filter clotting in the next 24 h. The combination of the cutoff values of urea CL/F (94%) and Mb CL/F (64%) showed a significant association with filter clotting after adjusting for confounding factors. The results obtained in this retrospective observational study can help avoid unexpected clotting, if confirmed by prospective interventional trials in the future.

## Figures and Tables

**Table 1:** Data at 24 h after CHDF initiation.

		Clotting Group *n* = 127	Non-Clotting Group *n* = 648	*p* Value
CHDF prescription	QF (mL/h)	534 ± 107	538 ± 154	0.7545
	QD (mL/h)	949 ± 270	905 ± 371	0.1189
	QB (mL/min)	94 ± 40	93 ± 16	0.6892
ACT (s)		154 ± 29	163 ± 35	0.0017
Anticoagulants	MN, *n* (%)	112 (88%)	545 (84%)	0.4092
	Heparin, *n* (%)	2 (1.6%)	34 (5.2%)	
	MN and heparin, *n* (%)	10 (7.9%)	48 (7.4%)	
	None, *n* (%)	3 (2.4%)	21 (3.2%)	
Anticoagulants dose	MN (mg/h)	30.1 ± 12.6	24.5 ± 13.5	0.0120
	Heparin (U/h)	456.7 ± 356.2	362.0 ± 140.1	0.3814
Filter	Polysurfone, *n* (%)	80 (63%)	347 (54%)	0.1512
	AN-69ST, *n* (%)	44 (35%)	284 (44%)	
	Others, *n* (%)	3 (2%)	15 (2%)	

QF, filtration rate; QD, dialysis flow rate; QB, blood flow rate; ACT, activated coagulation time; MN, nafamostat mesylate.

**Table 2:** ROC analysis of urea and Mb CL/F for predicting filter clotting.

	Clotting Group *n* = 127	Non-Clotting Group *n* = 648	AUROC (95% CI)	Cutoff	Sensitivity	Specificity
Urea CL/F	97.3 ± 8.4% *	99.7 ± 5.1%	0.56 (0.50–0.62)	97.0%	40.1%	76.6%
Mb CL/F	73.9 ± 19.5% *	82.5 ± 25.4%	0.61 ^#^ (0.55–0.66)	64.5%	37.8%	80.1%

* *p* < 0.05, versus non-clotting group. ^#^ *p* < 0.05.

**Table 3:** Patient characteristics and data of initial CHDF in each patient.

		Clotting Group *n* = 39	Non-Clotting Group *n* = 191	*p* Value
Age		74 (58–78)	66 (52–74)	0.1770
Sex (male), *n* (%)		30 (77%)	134 (70%)	0.3946
Background diseases, *n* (%)	Cardiovascular	31 (79%)	107 (56%)	0.0159953
	Sepsis	2 (5%)	38 (20%)	
	Others	6 (15%)	46 (24%)	
SOFA at CHDF initiation		12 ± 4	12 ± 3	0.7640
cardiovascular SOFA at CHDF initiation		2.5 ± 1.7	2.8 ± 1.6	0.2326
ACT at 24 h (s)		150 ± 25	161 ± 31	0.043
Type of membrane, *n* (%)	AN69ST	12 (31%)	97 (51%)	0.0517
	Polysurfone	27 (69%)	92 (48%)	
	Others	0	2 (1%)	

**Table 4:** ROC analysis of urea and Mb CL/F of the first CHDF in each patient.

	**Clotting Group** ***n* = 39**	**Non-Clotting Group** ***n* = 191**	**AUROC** **(95% CI)**	**Cutoff**	**Sensitivity**	**Specificity**
Urea CL/F	94.3 ± 11.1% *	100.0 ± 5.3%	0.63 ^#^ (0.52–0.75)	93.5%	38.5%	91.6%
Mb CL/F	70.4 ± 19.3% *	79.4 ± 21.8%	0.62 ^#^ (0.52–0.72)	64.5%	41.0%	77.5%

* *p* < 0.05, versus non-clotting group. ^#^ *p* < 0.05.

**Table 5:** Multiple regression analysis for clotting prediction within 24 h.

Variable	Odds Ratio (95% CI)	*p* Value
Urea CL/F < 94% and Mb CL/F < 64%	7.70 (2.28–26.1)	0.0010
Urea CL/F ≥ 94% or Mb CL/F ≥ 64% (reference)	1.00	
Background diseases		
Cardiovascular	2.00 (0.74–5.44)	0.1731
Sepsis	0.54 (0.10–2.94)	0.4793
Others (reference)	1.00	
ACT at 24 h	0.99 (0.98–1.01)	0.2267
Type of membrane		
AN69ST	0.69 (0.31–1.54)	0.3622
Others (reference)	1.00	

## Data Availability

The data presented in this study are available on request from the corresponding author.
